# Comparison of the Efficacy and Safety of a Pharmacokinetic Model-Based Dosing Scheme Versus a Conventional Fentanyl Dosing Regimen For Patient-Controlled Analgesia Immediately Following Robot-Assisted Laparoscopic Prostatectomy: A Randomized Clinical Trial

**DOI:** 10.1097/MD.0000000000002542

**Published:** 2016-01-15

**Authors:** Seok-Joon Jin, Hyeong-Seok Lim, Youn-Ju Kwon, Se-Ung Park, Jung-Min Yi, Ji-Hyun Chin, Jai-Hyun Hwang, Young-Kug Kim

**Affiliations:** From the Department of Anesthesiology and Pain Medicine (S-JJ, S-UP, J-MY, J-HC, J-HH, Y-KK); Department of Clinical Pharmacology and Therapeutics (H-SL); and Department of Nursing, Asan Medical Center, Seoul, Republic of Korea (Y-JK).

## Abstract

Conventional, intravenous, patient-controlled analgesia, which is only administered by demand bolus without basal continuous infusion, is closely associated with inappropriate analgesia. Pharmacokinetic model-based dosing schemes can quantitatively describe the time course of drug effects and achieve optimal drug therapy. We compared the efficacy and safety of a conventional dosing regimen for intravenous patient-controlled analgesia that was administered by demand bolus without basal continuous infusion (group A) versus a pharmacokinetic model-based dosing scheme performed by decreasing the dosage of basal continuous infusion according to the model-based simulation used to achieve a targeted concentration (group B) following robot-assisted laparoscopic prostatectomy.

In total, 70 patients were analyzed: 34 patients in group A and 36 patients in group B. The postoperative opioid requirements, pain scores assessed by the visual analog scale, and adverse events (eg, nausea, vomiting, pruritis, respiratory depression, desaturation, sedation, confusion, and urinary retention) were compared on admission to the postanesthesia care unit and at 0.5, 1, 4, 24, and 48 h after surgery between the 2 groups. All patients were kept for close observation in the postanesthesia care unit for 1 h, and then transferred to the general ward.

The fentanyl requirements in the postanesthesia care unit for groups A and B were 110.0 ± 46.4 μg and 77.5 ± 35.3 μg, respectively. The pain scores assessed by visual analog scale at 0.5, 1, 4, and 24 h after surgery in group B were significantly lower than in group A (all *P* < 0.05). There were no differences in the adverse events between the 2 groups.

We found that the pharmacokinetic model-based dosing scheme resulted in lower opioid requirements, lower pain scores, and no significant adverse events in the postanesthesia care unit following robot-assisted laparoscopic prostatectomy in comparison with conventional dosing regimen.

## INTRODUCTION

Laparoscopic surgery may induce more severe pain in comparison with open laparotomy due to inappropriate pain management during the immediate postoperative period.^1^ As pain management usually starts after admission to the postanesthesia care unit, a gap between the recognition of pain and inducing analgesia using demand doses of intravenous patient-controlled analgesia (PCA) or additional bolus doses of opioid can exist. Therefore, conventional dosing regimens for intravenous PCA,^2^ which are performed by intermittent bolus administration without the basal continuous infusion of opioids, may exhibit inadequate analgesic effects during the immediate postoperative period in patients in the postanesthesia care unit who have undergone robot-assisted laparoscopic prostatectomy (RALP).

The pharmacokinetic characteristics of fentanyl include its short duration of action, high lipid solubility, rapid distribution to fat and skeletal muscle, and a high extraction ratio drug.^3^ Together with these properties, its tolerability and wide therapeutic range make fentanyl a suitable opioid for PCA.^2^ In terms of the respiratory effects of fentanyl, the basal continuous infusion of 0.12 to 0.67 μg/kg/h fentanyl does not result in significant respiratory depression.^4,5^ However, the basal continuous infusion of intravenous PCA is not usually recommended for postoperative pain management due to the risk of opioid accumulation and concerns about respiratory depression.^6^ As respiratory depression is closely related to high plasma concentrations of fentanyl, which may be caused by not only continuous infusion but also by high bolus doses,^7,8^ target-concentration strategies may be safer and more effective than conventional dosing regimens. However, little is known about the efficacy and safety of pharmacokinetic model-based fentanyl dosing schemes, which can quantitatively describe the time course of drug effects and achieve optimal drug therapy,^9^ for adequate pain management following RALP.

In our present study, we compared the opioid requirements in the postanesthesia care unit, visual analog scale (VAS) scores for pain, and adverse events during the postoperative period between a conventional dosing regimen and pharmacokinetic model-based dosing scheme in patients who underwent RALP.

## MATERIALS AND METHODS

### Patient Characteristics and Study Procedure

The protocol was approved by the institutional review board of Asan Medical Center, Seoul, Republic of Korea (approval number: 2015-0267), and written informed consent was obtained from patients with prostate cancer who underwent RALP between April 2015 and September 2015. This study was registered with the ClinicalTrials. gov database (NCT02402621). Exclusion criteria included allergies to opioids, history of chronic pain, history of alcohol or drug abuse, history of sleep apnea or respiratory complications, American Society of Anesthesiologists physical status ≥ 3, age < 20 years or ≥ 70 years, and body mass index > 30 kg/m^2^. Patients who had taken opioids or analgesics before surgery were also excluded. Before surgery, all patients were instructed how to use the PCA pump and VAS for pain scoring by our acute pain service team. No patients were premedicated before surgery.

After applying a routine monitoring system (electrocardiography, pulse oximetry, invasive blood pressure, and bispectral index), general anesthesia was induced using a bolus intravenous injection of 5 mg/kg thiopental sodium and 0.6 mg/kg rocuronium and maintained using 5 to 6 vol% desflurane plus an effect-site target-controlled infusion 2 to 5 ng/mL remifentanil with 50% oxygen in medical air. Patients were mechanically ventilated at a constant tidal volume of 6 to 8 mL/kg, and the respiratory rate was adjusted to maintain the end-tidal carbon dioxide partial pressure between 35 and 40 mm Hg during the operation.

**FIGURE 1:**
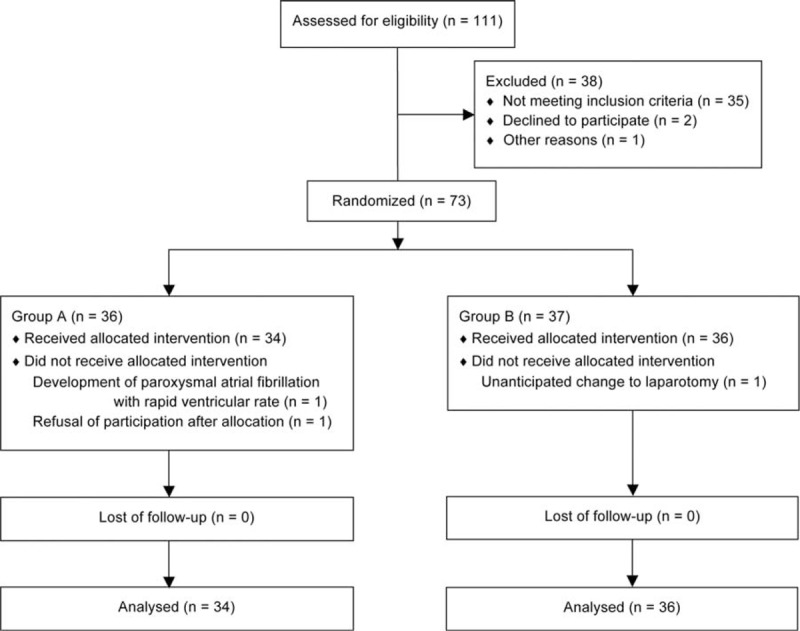
CONSORT flow diagram.

**FIGURE 2:**
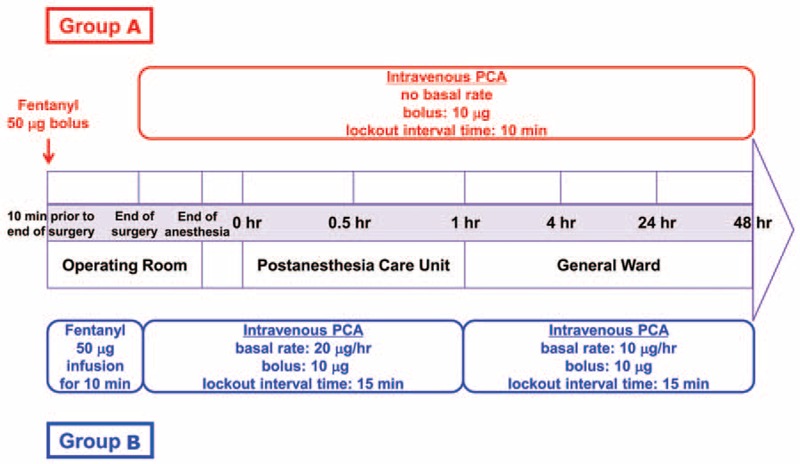
Schematic representation of the study protocol. PCA = patient-controlled analgesia.

Patients were randomly allocated to receive the conventional dosing regimen performed by intermittent bolus administration without basal continuous infusion (group A) or the pharmacokinetic model-based dosing scheme performed by decreasing the dose of basal continuous infusion according to the model-based simulation in order to achieve a targeted concentration (group B) (Figure 1). Figure 2 shows a schematic representation of the study protocol. Ten minutes before the completion of surgery, the patients in groups A and B received 50 μg fentanyl via intravenous bolus injection or 50 μg fentanyl via continuous infusion for 10 min, respectively. Upon the completion of surgery, the patient was extubated after recovery from rocuronium using 2 mg/kg sugammadex delivered via intravenous bolus injection. In group A, patients received PCA, which consisted of a bolus of 10 μg fentanyl with a 10-min lockout interval without basal continuous infusion at the end of surgery; in group B, patients received PCA, which consisted of a bolus of 10 μg fentanyl with a 15-min lockout interval with the basal continuous infusion of 20 μg/h fentanyl for 1 h, and thereafter the rate of basal continuous infusion was reprogrammed to 10 μg/h fentanyl according to the results of the pharmacokinetic simulation of fentanyl.^3,10^

In the postanesthesia care unit, if the VAS score (0 was defined as no pain, and 10 was defined as the worst pain ever experienced) of the patient was ≥ 6,^11,12^ which indicates severe pain, the physician intravenously administered a bolus of 25 μg fentanyl. The opioid requirements in the postanesthesia care unit were measured. All patients were kept for close observation in the postanesthesia care unit for 1 h and then transferred to the general ward. VAS scores and adverse events (eg, nausea, vomiting, pruritis, respiratory depression, desaturation, sedation, confusion, and urinary retention) were measured on admission to the postanesthesia care unit and at 0.5, 1, 4, 24, and 48 h after surgery by an investigator who was blind to the treatment groups. The definitions of respiratory depression, desaturation, and sedation were a respiratory rate < 12 breaths/min, oxygen saturation < 90%, and level of sedation so that the patient has a normal response to verbal stimuli, respectively. The total fentanyl dose administered during the postoperative period was calculated by combining the infused fentanyl doses and any other opioids or analgesics that were administered during the postoperative period. All opioids and analgesics were recorded and converted to fentanyl-equivalent doses using morphine sulfate equivalents.^13–15^ The total duration of surgery (from the start of surgery to the end of surgery in minutes) and time to extubation from the end of surgery (from the end of surgery to extubation in minutes) were also recorded.

### Computer Simulations

**FIGURE 3:**
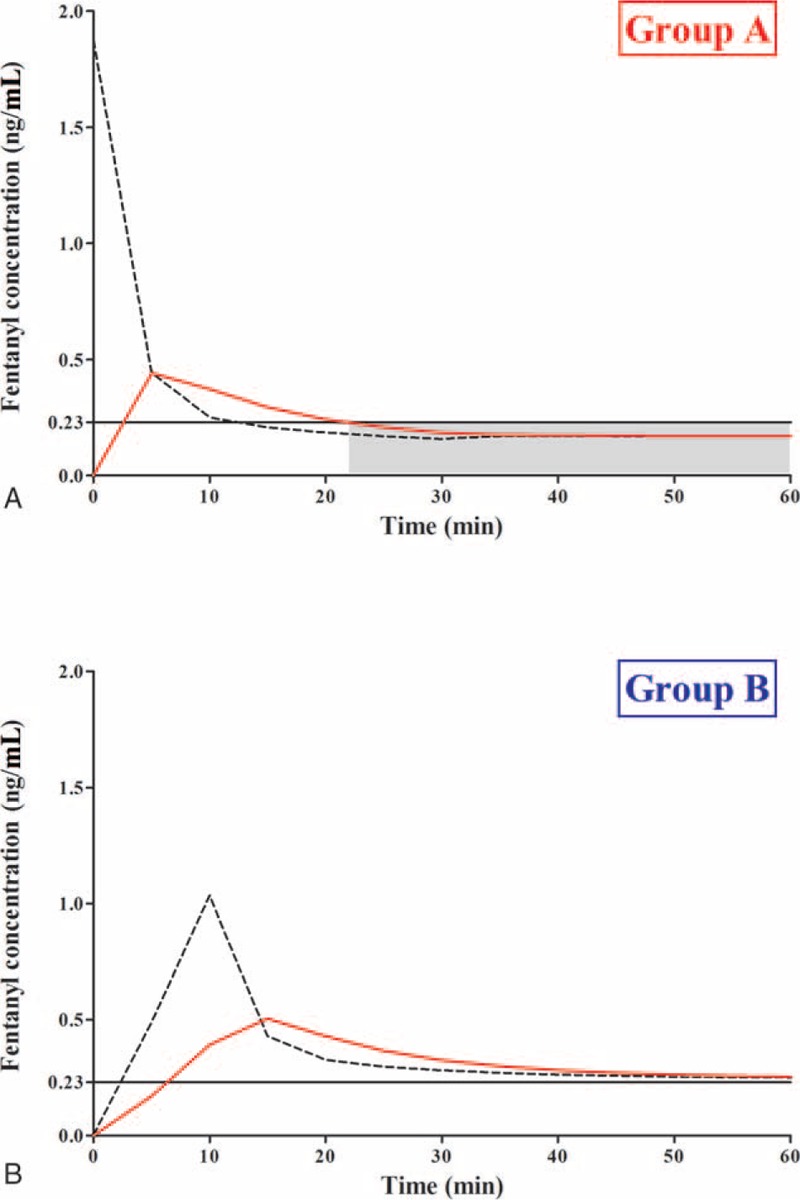
Simulated plasma concentrations of fentanyl over time in groups A (A) and B (B). The black dash lines and red solid lines represent the plasma and effect-site concentrations of fentanyl, respectively. The black horizontal solid lines (Y = 0.23 ng/mL) represent the minimal effect concentrations of fentanyl. The gray shadow box in group A represents the undertreated period. In contrast, the effect-site concentration in group B did not decrease to below the minimal effect concentration of fentanyl until after 60 min.

The optimal dosing scheme for model-based PCA infusion—that is, maintaining the target concentration above the minimal effective concentration of 0.23 ng/mL^16^ and below the concentration that can result in unwanted side effects^4^—was determined according to the pharmacokinetic parameters of fentanyl as follows:^3,10^*V*_central_ (L): 26.6, *k10* (min^–1^): 0.0332, *k12* (min^–1^): 0.172, *k13* (min^–1^): 0.131, *k21* (min^–1^): 0.1, *k31* (min^–1^): 0.0177, and *Ke0* (min^–1^): 0.147. In this present study, we determined the targeted concentration, which is slightly higher than the minimal effective concentration throughout the postoperative period, and then simulated the cumulative drug amount in order to maintain these concentrations using NONMEM^®^ VII level 2 (ICON Development Solutions, Dublin, Ireland). After simulating the time-concentration curve using various dosing schemes, we selected the optimal dosing regimen that could maintain the appropriate concentrations and entered the basal rate into the intravenous PCA infusion pump (AutoMed 3200^®^; Ace Medical Co., Seoul, Republic of Korea). Deterministic simulations using point estimates of the fixed-effect parameters were performed to provide an illustration of the predicted time courses of the plasma and effect-site concentrations after fentanyl administration. Variances in the random-effect parameters (interindividual and residual) were set to 0. Figure 3 shows the simulated plasma and effect-site concentrations of fentanyl over time for 2 different practical situations that represent groups A and B.

### Statistical Analysis

The sample size was estimated based on the power calculation, which showed that ≥33 patients per group were necessary to achieve 80% power and detect a 20% difference between computer-controlled alfentanil infusion and conventional PCA morphine infusion in order to reduce opioid requirements.^17^ The total estimated number of patients was 73 after considering a 10% dropout rate. Data such as demographics, surgical duration, time to extubation from the end of surgery, opioid requirements, and VAS score were compared with using Student's *t* test or Mann–Whitney *U* test, as appropriate. The American Society of Anesthesiologists physical status and incidences of adverse events were measured by using the χ^2^ test or Fisher's exact test, as appropriate. Statistical analysis and randomization were conducted using R (version 3.1.2; R Foundation for Statistical Computing, Vienna, Austria), SigmaStat 3.5 for Windows (Systat Software, Inc., Chicago, IL), and SPSS 22 for Windows (version 22.0.0; IBM Corporation, Chicago, IL). Data are expressed as the mean ± SD, or number (percentage) as appropriate. In this study, *P* < 0.05 was considered statistically significant.

## RESULTS

This study included 73 patients who underwent RALP. After randomization, however, 3 patients were not included in the analysis due to the development of intraoperative paroxysmal atrial fibrillation with a rapid ventricular rate, refusal to participate in the trial just before induction, or an unanticipated change to laparotomy due to severe peritoneal adhesion, respectively (Figure 1).

**FIGURE 4:**
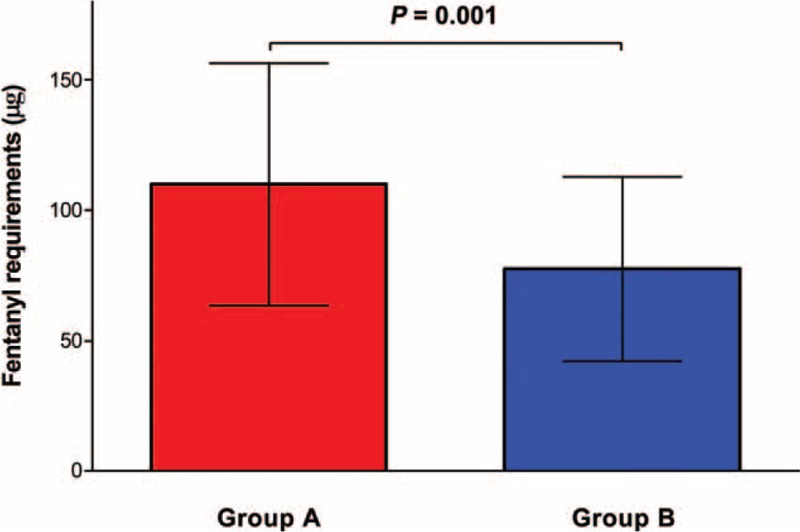
Comparison of the fentanyl requirements in the postanesthesia care unit between group A (red box) and group B (blue box) in patients who underwent robot-assisted laparoscopic prostatectomy. The fentanyl requirement in group B was significantly lower in comparison with group A. The upper borders of the box and error bars of each group represent the mean and SD, respectively.

The patient characteristics and outcomes for groups A and B are compared in Table 1. The fentanyl requirements in the postanesthesia care unit in groups A and B were 110.0 ± 46.4 μg and 77.5 ± 35.3 μg, respectively (Figure 4). The VAS scores of groups A and B at each time point are also compared in Table 1. The VAS scores at 0.5, 1, 4, and 24 h after surgery in group B were significantly lower than in group A. Adverse events were not significantly different between the groups (Table 1). The total fentanyl doses administered during the postoperative period to groups A and B were 589.5 ± 333.3 μg and 890.4 ± 200.1 μg, respectively.

**TABLE 1:**
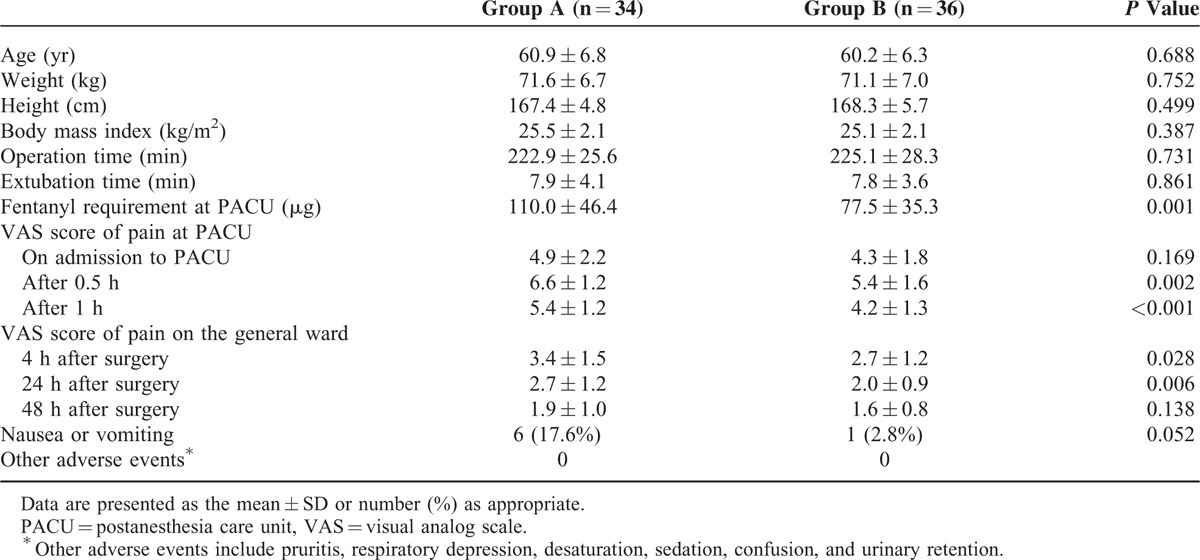
Patient Characteristics and Outcomes

## DISCUSSION

Among our patients who underwent RALP, the pharmacokinetic model-based dosing scheme for fentanyl PCA demonstrated significant reductions in opioid requirements in the postanesthesia care unit and pain scores assessed by VAS during the postoperative period without developing any significant opioid-related complications in comparison with the conventional dosing regimen. Our data suggest that both the plasma and effect-site concentrations did not decrease below the minimal effective concentration of fentanyl in the model-based PCA group. However, in the conventional PCA group, we believe the fentanyl concentration decreased to below the minimal effective concentration around admission to postanesthesia care unit, which may have resulted in moderate to severe pain.

Fentanyl is a highly lipophilic drug, which facilitates its distribution into the brain, and is well suited for intravenous PCA. Furthermore, using fentanyl for intravenous PCA demonstrates considerable analgesic efficacy with good tolerability.^18–20^ Although fentanyl has a wide therapeutic window, large on-demand doses or high basal infusion rates are associated with the risk of respiratory depression.^21,22^ In addition, the long context-sensitive half-time of fentanyl after a long period of infusion results in a dramatic increase in its concentration, which may be associated with postoperative complications such as respiratory depression, nausea, and vomiting.^23,24^ For these reasons, basal continuous infusion is not routinely recommended as a postoperative analgesia.

However, pain after laparoscopic surgery is known to be more intense during the immediate postoperative period in comparison with after open laparotomy.^1^ Furthermore, in our present study, the administration of only the demand dose of fentanyl—without basal continuous infusion—demonstrated inadequate pain relief in the postanesthesia care unit in patients who underwent RALP. Similar to the previous simulation results, the fentanyl concentration progressively decreased to the minimal effective concentration at ∼30 min after the first fentanyl bolus injection. This time course of the concentration was inversely related to the trends of the pain scores, which increased abruptly after admission to the postanesthesia care unit and peaked at 30 min after admission. In addition, the simulated fentanyl concentration of the demand dose plus an additional fentanyl bolus in the postanesthesia care unit showed not only an abrupt increase in the fentanyl effect-site concentration to > 2 ng/mL—which may produce fentanyl-related complications—but also that the fentanyl concentration could have decreased to below the minimal effective concentration of 0.23 ng/mL and thereby resulted in inappropriate pain management. Considering the appropriate dosing scheme for managing postoperative analgesia, there are problematic concerns regarding the lack of basal continuous infusion with a demand bolus dose, which may have led to an insufficient fentanyl concentration below the minimal effective concentration.

Taken together, we adapted pharmacokinetic model-based PCA as an advanced method for reducing the period during which the fentanyl concentration is below the minimal effective concentration during the postoperative period. We found that the model-based dosing scheme reduced analgesic requirements in the postanesthesia care unit and pain scores during the postoperative period among patients who underwent RALP. Pharmacokinetic model-based dosing schemes, which can provide a quantitative description of the time course of drug effects and offer great potential for achieving optimal drug therapy, are increasingly used within most therapeutic areas.^9^ The model-based dosing scheme is very popular in drug development area, especially in first-in-human studies and trials on optimal dosing regimens.^25,26^ In addition, these approaches helped to achieve successful registration of the drug and were identified as a more efficient trial design that reduced the required number of subjects and thereby saved time. Therefore, model-based PCA, which can restrict concentrations within the therapeutic range, can provide tailored postoperative analgesic management approaches.

In terms of study limitations, we did not examine the predictive performance of the simulated PCA regimen, which should take blood samples at predetermined time points. In addition, our simulation did not include interindividual variations in the pharmacokinetic parameters of fentanyl. However, the pharmacokinetic model of fentanyl is largely accepted to be valid for all patients,^27^ except children and overweight patients.^28,29^ Therefore, our results are promising for the postoperative pain management of patients who undergo RALP.

In conclusion, pharmacokinetic model-based PCA, which can provide optimal basal infusion in order to achieve a concentration above the minimal effective concentration, significantly decreases the opioid requirements and pain scores without inducing serious adverse events immediately following RALP. These results provide a better understanding of effective pain management following RALP.
